# Return to work of breast cancer survivors: toward an integrative and transactional conceptual model

**DOI:** 10.1007/s11764-021-01053-3

**Published:** 2021-05-05

**Authors:** Bertrand Porro, Marie-José Durand, Audrey Petit, Mélanie Bertin, Yves Roquelaure

**Affiliations:** 1 Univ Angers, Univ Rennes, Inserm, EHESP, IRSET (Institut de Recherche en Santé, Environnement et Travail) - UMR_S 1085, SFR ICAT, F-49000 Angers, France; 2 Centre d’action en prévention et réadaptation des incapacités au travail (CAPRIT), Université de Sherbrooke, 150 Place Charles-Le Moyne, Suite 200, Longueuil, QC J4K 0A8 Canada; 3 Centre de recherche Charles-Le-Moyne-Saguenay-Lac-Saint-Jean sur les innovations en santé (CR-CSIS), Université de Sherbrooke, 150 Place Charles-Le Moyne, Suite 200, Longueuil, QC J4K 0A8 Canada; 4 School of Rehabilitation, Faculty of Medicine and Health Sciences, Université de Sherbrooke, 150 Place Charles-Le Moyne, Suite 200, Longueuil, QC J4K 0A8 Canada; 5 Univ Angers, CHU Angers, Univ Rennes, Inserm, EHESP, IRSET (Institut de Recherche en Santé, Environnement et Travail) - UMR_S 1085, SFR ICAT, F-49000 Angers, France; 6 Univ. Rennes, EHESP, REPERES (Recherche en pharmaco-épidémiologie et recours aux soins) – EA 7449, F-35000 Rennes, France

**Keywords:** Breast cancer survivors, Return to work, Conceptual model, Determinants, Expert consensus, TRIAGE method

## Abstract

**Purpose:**

To propose a conceptual framework of the return to work (RTW) of breast cancer survivors (BCS) according to the transactional perspective.

**Methods:**

The Technique for Research of Information by Animation of a Group of Experts was implemented. For each determinant in an initial list established from the literature, experts selected for the consensus exercise were firstly asked to indicate their agreement level individually, via an online questionnaire. Determinants obtaining an agreement level of 80% or over during this first phase were retained. Determinants obtaining an agreement level below 80%, and additional determinants proposed by the experts, were then discussed collectively. After discussion, experts voted via a new online questionnaire to retain (or not) each determinant. Determinants obtaining an agreement level of 80% or over after this second phase were retained. Based on the determinants selected, a conceptual model was developed following the transactional approach.

**Results:**

Eleven experts participated in the study. Forty of the 51 determinants listed initially from the literature achieved an agreement level over 80%, and 20 were added after the individual consultation phase. Twenty-two of the 31 determinants discussed collectively were retained. In total, 62 determinants were selected to construct the conceptual model.

**Conclusions:**

This integrative, operational, and transactional conceptual model of the RTW of BCS, constructed following an expert consensus, will help to design more efficient patient-centered intervention studies.

**Implications for Cancer Survivors:**

Identification of the 62 determinants associated with the RTW of BCS will help design tools that are easily used by all stakeholders involved in the RTW process.

## Introduction

Breast cancer (BC) is the most common cancer diagnosis among women worldwide [1]. Progress in both the effectiveness of prevention campaigns and treatments have reduced cancer mortality [1, 2]. BC survivors (BCS) must therefore live with the long-term side effects of cancer and its treatment (e.g., fatigue, pain, or emotional distress) that can significantly affect the working lives of those of working age [3, 4]. Return to work (RTW) after BC is presented as a desirable outcome, both from individual and social points of view [5–7]. For BCS, a successful RTW is essential for regaining a sense of normalcy, improving self-esteem, providing financial security, maintaining social relationships, and restoring functional abilities [7–9]. Each BCS is unique and is at the heart of her RTW process which involves many stakeholders [10]. A better understanding of the RTW process is thus essential in order to propose appropriate interventions aimed at facilitating the RTW of BCS and its sustainability.

The main methodological criticisms of interventions that promote the RTW of cancer patients are the lack of (i) theory-based interventions [11, 12], (ii) knowledge of the determinant(s) that need to be addressed [11], and (iii) patient-centered interventions [11, 13]. Knauf and Schultz [14] highlighted a need for a transdisciplinary model of RTW that addressed the temporal and multidimensional aspects of disability. The proposal of such a model would help to provide both a better understanding of the RTW process in clinical practice and to frame interventions that promote RTW.

The development of a conceptual model requires a clear definition of the outcome, whereas a clear definition of RTW remains elusive [14, 15]. According to the BC literature, there is general confusion between the terms *RTW*, *return to employment*, and *staying at work* [4, 16]. An attempt to clarify these definitions follows: *RTW* can be defined as the process of returning to the same work situation in place pre-diagnosis after a full period of sick leave (with or without accommodation). A *return to employment* involves three possible scenarios: (i) the patient has experienced a contractual break with work (e.g., job loss; the ending of a fixed-term contract) and returns to a new professional situation; (ii) the patient undergoes internal reclassification within the same company or administration due to her inability to resume her former role because of her new health issues (e.g., disability, restricted arm movement); and (iii) the patient undergoes external reclassification and returns to a new professional situation in a new company or administration. After an efficient RTW (or an efficient return to employment), stakeholders (i.e., patient, medical practitioner, and employer/managers) try to implement all means necessary to ensure a *sustainable RTW* (or *sustainable return to employment*). *Staying at work* concerns patients who do not take a full period of sick leave because of BC (i.e., part-time sick leave or no sick leave).

A conceptual model must be theory-based [14], whereas four conceptual models available in the literature are not [8, 17–19]. According to Schultz et al. [20] and Knauf and Schultz [14] the biopsychosocial perspective seems to be most appropriate to describe, in an interdisciplinary manner, the processes involved in RTW (i.e., the relationships between the different determinants of RTW). Despite its comprehensiveness, the biopsychosocial perspective has been widely criticized as being linear, juxtaposing the biological, psychological, and social parameters that impact health outcomes without real integration [21]. Occupational outcomes after cancer are characterized by complex relationships between several sociodemographic, medical, professional, economic, psychosocial, and behavioral determinants [4, 5, 16]. RTW should not be seen as a static interactional process since determinants change over time and need to take account of worker expectations [14, 22, 23]. Another perspective takes into account all these factors while stressing their dynamic and temporal relationships: the transactional perspective [24]. The transactional perspective postulates constant adaptation between individuals and their environment [24], with a primary appraisal of the situation (perceived as a loss, a threat, or a challenge) and a secondary appraisal of personal (e.g., ability to control) or environmental (e.g., perceived social support) resources. The transactional perspective conceptualizes all strategies implemented to deal with the situation as coping strategies (problem-centered coping, emotion-centered coping, or benefit finding) which mediate the relationships between appraisals and outcomes [24, 25]. A dynamic perspective is also taken into account by systematic re-evaluation of the situation (feedback) [24]. The emergence of an integrative, multifactorial, and dynamic model, based in particular on the transactional perspective, could allow a better understanding of the dynamic process of the RTW of BCS.

A multidisciplinary conceptual model of RTW could also introduce knowledge of the ergonomics of activities, such as “the margin of manoeuvre” (MM) that is pivotal to the RTW process [14, 26, 27]. MM is defined as the possibility that a worker may develop different ways of working in order to meet production targets, without affecting their health [27]. Three types of MM are involved in the RTW process [27]: (i) *the initial MM* that refers to an assessment of the worker’s initial work situation before the BC diagnosis; (ii) the *therapeutic MM* that corresponds to the development of individual or professional strategies to facilitate the RTW; and (iii) the final MM that must be sufficient to favor a sustainable RTW [27]. Studies focusing on patients with musculoskeletal disorders have shown associations between the MM and RTW [26, 27]. The concept of MM has not yet been identified in the literature dealing with RTW after BC, whereas several indicators of MM have been reported as displaying a connection with the RTW of BCS (e.g., professional support, job demands, functional capacities, or RTW self-efficacy) [27, 28].

A conceptual model of RTW must be evidence-based [14]. Systematic reviews have highlighted sociodemographic factors (e.g., age, socioeconomic status, level of education), medical factors (e.g., chemotherapy, higher stage BC), occupational factors (e.g., high psychological or physical work demands), and psychosocial or physical factors (e.g., pain, perceived social support, emotional distress, cancer-related fatigue) as determinants of the (non-)RTW of BCS [4, 16, 29]. It is also likely that some determinants, related to clinical practice or patient experience, may have not yet been identified in the published scientific literature while they may have a real impact on the (non-)RTW of BCS. A qualitative study, conducted by Tiedtke et al. [10], has shown the value of collecting the opinions of different stakeholders on RTW. Comparing the opinions of experts should lead to a broader identification of the determinants of the RTW of BCS [30].

A newly developed conceptual model of the RTW of BCS could serve as a framework for exploratory studies, interventional studies, and clinical practice, by explaining the articulation between the determinants involved. The objective of this study was to propose an integrative conceptual model of the RTW in BCS according to the transactional perspective, identifying the several determinants to be included by means of an expert consensus method.

## Methods

### Design

The Technique for Research of Information by Animation of a Group of Experts (TRIAGE) was implemented [31, 32]. TRIAGE is a dynamic decision-making technique based on a constructivist perspective which assumes that a consensus is constructed collectively [31, 32]. This technique differs from traditional methods (Delphi and Nominal Group techniques) in that it favors the creation of a common opinion via discussion [32]. The opinions of experts are requested twice: once individually and once collectively [31, 32]. TRIAGE can be described in four successive phases: (i) preparation; (ii) individual consultation; (iii) data compilation; and (iv) collective consultation [31, 32]. Because of COVID-19, two phases (data compilation and collective consultation) were modified while fully complying with the initial methodology [31, 32].

### Procedure

#### Preparation

This first preparatory step lasted 9 months (May 2019–January 2020). It included the recruitment of experts for the study and the identification of an initial list of the determinants of RTW in BCS.

##### Recruitment of experts for the study

The expert group had to include between six and twelve participants [31, 32]. We contacted different BC and RTW stakeholders (general practitioners, occupational practitioners, oncologists, psychologists, human resources managers, oncological nurses, researchers, social workers, lawyers, heads of patient associations, and expert patients) to compare as many opinions as possible. All experts were contacted by e-mail to request participation and the contact details of another expert in the field.

##### Initial list of the determinants of RTW in BCS

The development of the initial list of determinants of RTW in BCS was based on a PhD thesis that aimed at identifying the determinants of RTW in BCS [33] supplemented by a review of reviews focusing specifically on the determinants of RTW in BCS, published in English or French, up to 31 January 2020. Reviews that focused on outcomes other than RTW, on other types of cancer, or on interventions aiming at enhancing the RTW of BCS were excluded. Critical reviews were also excluded. The data extracted was limited to the major determinants identified in the literature reviews. The review was performed on PubMed and PsycINFO databases using the following algorithms:

- For PubMed: (("breast cancer"[Title]) AND ("return*"[Title] OR "work"[Title] OR "return to work"[Title] OR "sickness absence"[Title])) AND ("review"[Title] OR "meta-analysis"[Title] OR "meta-synthesis"[Title])
- For PsycINFO: TI "breast cancer" AND TI ( "return*" OR "work*" OR "return to work" OR "sickness absence" ) AND TI ( "review" OR "meta-analysis" OR "meta-synthesis" )

#### Individual consultation phase

The individual consultation phase lasted for 3 weeks (February 2020). Each expert responded individually to an online questionnaire. For each determinant, the experts had to give their opinion on the determinant of RTW in BCS selected previously through the literature review using a four-point Likert scale (1 Strongly disagree; 2 Disagree; 3 Agree; 4 Strongly agree). Each expert also had the opportunity to propose up to three supplementary determinants according to their experience (personal or professional), their clinical practice, or their scientific knowledge.

#### Data compilation

Data compilation (March 2020) consisted (i) in identifying the items on which consensus was reached during the individual consultation phase and (ii) in listing the additional determinants proposed by the experts in the individual consultation phase. For each BCS RTW determinant assessed, a first consensus was reached when 80% or more [31, 32] of the expert group selected “Agree”/“Strongly agree,” and this determinant was directly selected for development of the model. Determinants that did not achieve consensus at this first stage, in addition to the supplementary determinants proposed by the experts, were discussed during the collective consultation phase.

#### Collective consultation phase

The collective consultation phase was carried out in the presence of a trained moderator (PB) supported by two assistants (YR and AP). During this collective consultation phase (June 2020), experts had to discuss and then decide, by consensus, whether or not the determinants that did not achieve the 80% threshold, and those that were additionally proposed in the individual consultation phase, should be included. The collective consultation phase was carried out by videoconference in two three hours sessions that were audiotaped after the agreement of all participants. Each expert had to say whether they agreed or disagreed with the retention of a specific determinant by advancing one line of reasoning. All arguments were noted on an online visual tool. Once the opinions of all participants had been collected, the whole group was allowed several minutes to discuss the determinant in question. The moderator had to ensure that everyone participated. At the end of the discussion, experts had to vote, individually and anonymously, via a new online questionnaire, on whether they agreed or disagreed with retaining the determinant under discussion (i.e., “Yes” or “No”). All determinants that received 80% or more “Yes” votes were retained. Determinants that did not receive 80% “Yes” votes were excluded. The results were forwarded immediately to the experts.

### Development of an integrative and transactional conceptual model

Once the results of the TRIAGE exercise were obtained, each determinant was classified by the first (PB) and the last (YR) authors, according to the antecedents and the broad categories of the transactional perspective (primary assessment, secondary assessment, adjustment strategies) [24]. A first version of the model was proposed to the co-authors (MJD, MB, AP) for review. After corrections, the conceptual model was presented to the TRIAGE group of experts in several working meetings according to the method of Le Boutiller et al. [34]. Owing to COVID-19 and personal obligations, it was not possible to reconvene a meeting of all experts at the same time. They were asked to comment orally on the general language and the positioning of concepts within the different categories of the conceptual model. The conceptual model was modified in response to these comments, to produce the final conceptual model.

## Results

### Preparation

#### Recruitment of experts for the study

Twenty-four experts were contacted of which seven (four human resources managers, two oncologists, and one occupational psychologist) did not reply, five (an oncologist, a social worker, a human resources manager, an epidemiologist, and an expert patient) refused because of lack of time, and 12 agreed to participate. Of the remaining 12 experts, one expert patient left the procedure at the individual consultation phase for personal reasons. The final sample consisted of 11 experts whose characteristics are presented in Table 1. The expert group consisted of four researchers, four physicians, two psychologists, two expert patients, one human resources manager, one lawyer, one nurse, and one social worker (Table 1). Nine were BC specialists, nine were RTW specialists, and seven were RTW after BC specialists (Table 1).

**Table 1:** Expert group characteristics

Expert	Gender	Roles	Expertise in breast cancer	Expertise in return to work
1	Male	Occupational practitioner Senior researcher	X	X
2	Male	Health psychologist Researcher	X	X
3	Female	Occupational practitioner Senior researcher	X	X
4	Female	General practitioner Researcher	X	X
5	Female	Lawyer Association head	X	X
6	Female	Oncological Nurse	X	
7	Female	Expert patient	X	
8	Female	Occupational psychologist Human resources manager		X
9	Female	Social worker		X
10	Female	Oncologist Occupational practitioner	X	X
11	Female	Expert patient Association head	X	X

#### Initial list of the determinants of RTW in BCS

**Fig. 1:**
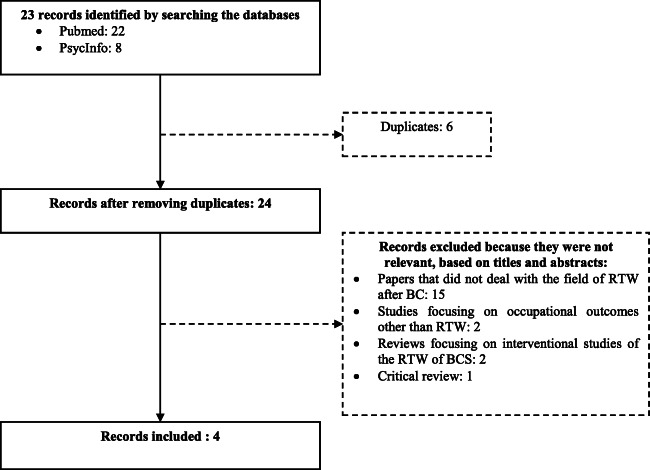
Flowchart of the article selection process

In addition to the PhD thesis [33], the review identified four articles that met our inclusion criteria, [4, 35–37]. Figure 1 shows a flowchart of the article selection process. A list of the 51 proposed determinants is presented in Table 2.

**Table 2:** Results of individual consultation phase

Determinants	Insertion		To be discussed
	100% consensus	≥ 80% consensus	< 80% consensus
**Sociodemographic factors**			
Age	X		
Education		X	
Dependent children	X		
Ethnicity			X
Place of residence			X
Marital status			X
**Professional factors**			
Socio-professional category		X	
Professional status	X		
Type of contract	X		
Seniority in the company		X	
Hierarchical position	X		
Business sector			X
**Financial factors**			
Income		X	
Wage loss	X		
**Medical factors**			
Cancer stage		X	
Type of surgery	X		
Chemotherapy		X	
Radiation therapy	X		
Hormone therapy		X	
Two or more cancer diagnoses			X
**Values**			
Intention to RTW	X		
Meaning of work		X	
Work attachment		X	
**Physical health**			
Global health status		X	
Physical fatigue	X		
Cognitive fatigue	X		
Emotional fatigue		X	
Pain	X		
Physical sequelae	X		
**Psychological factors**			
Emotional distress		X	
Depression		X	
Anxiety		X	
RTW self-efficacy		X	
Benefit-finding		X	
Post-traumatic growth			X
Problem-focused coping			X
Emotion-focused coping			X
Optimism			X
Life satisfaction			X
**Work environment**			
Physical stressors at work	X		
Psychological stressors at work	X		
Organizational stressors at work	X		
Work-related stress	X		
Perceived social support from colleagues	X		
Perceived social support from managers	X		
Opportunity for career advancement			X
**Personal environment**			
Perceived social support from family	X		
Perceived social support from friends		X	
**Therapeutic margin of manoeuvre**			
Working time accommodation	X		
Workstation accommodation	X		
Professional duties accommodation	X		

*Notes.* No determinant was rejected

### Individual consultation phase

Of the 51 determinants evaluated in the individual consultation phase, 40 determinants achieved consensus. Twenty-three factors achieved 100% consensus for insertion and 17 factors achieved a consensus for insertion equal to or greater than 80% (Table 2). Eleven determinants required discussion in the collective consultation phase (below the 80% agreement threshold) (Table 2).

Twenty additional determinants were suggested during the individual consultation phase including “gender”, “social precariousness”, “company size”, “being the main family breadwinner”, “health insurance”, “immunotherapy”, treatment with “trastuzumab”, “sense of professional usefulness”, “relationship to work”, “disability due to BC”, “restricted arm movement”, “body image”, “recognition by colleagues of the quality of work performed”, “recognition of the BCS by line management in her professional activity”, “maintenance of contact with colleagues during sick leave”, “quality of the met supportive care during BC treatments”, “perceived social support from medical staff”, “early liaison with occupational practitioner or occupational health department”, “social worker support”, and “manner in which the patient was informed of the BC diagnosis” (i.e., physician’s empathy).

### Collective consultation phase

One expert was unable to attend the collective consultation phase for technical reasons (expert 10; Table 1). Of the remaining 31 determinants discussed (11 that did not achieve a consensus in the individual consultation phase + 20 additional determinants from the individual consultation phase), 22 were retained.

Of the 11 factors on which there was no consensus during the individual consultation phase, six were selected in the collective consultation phase, of which two achieved 100% acceptance (i.e., “place of residence,” “two or more cancer diagnoses”) and four achieved 90% acceptance (i.e., “ethnicity”, “post-traumatic growth”, “problem-focused coping”, “emotion-focused coping”).

Of the 20 additional factors proposed during the individual consultation phase, 16 were selected in the collective consultation phase of which 13 achieved 100% acceptance (i.e., “social precariousness”, “company size”, “being the main family breadwinner”, “health insurance”, “immunotherapy”, “treatment with trastuzumab”, “sense of professional usefulness”, “disability due to BC”, “restricted arm movement”, “body image”, “recognition of the BCS by line management in her professional activity”, “perceived social support from medical staff”, “early liaison with occupational practitioner or occupational health department”), two achieved 90% acceptance (i.e., “recognition by colleagues of the quality of work performed”, “social worker support”), and one achieved 80% acceptance (i.e., “quality of the met supportive care during BC treatments”).

The determinants rejected were “gender”, “marital status”, “manner in which the patient was informed of the BC diagnosis”, “business sector”, “opportunity for career advancement”, “life satisfaction”, “optimism”, “maintenance of contact with colleagues during sick leave”, and “‘relationship to work”. Finally, a total of 62 determinants were selected (Table 3).

**Table 3:** Final 62 determinants selected by the expert group

**Sociodemographic factors**	
1	Age
2	Education
3	Ethnicity
4	Place of residence
5	Dependent children
6	Social precariousness
**Professional factors**	
7	Socio-professional category
8	Professional status
9	Company size
10	Type of contract
11	Seniority in the company
12	Hierarchical position
**Financial factors**	
13	Income
14	Being the main family breadwinner
15	Wage loss
16	Health insurance
**Medical factors**	
17	Cancer stage
18	Two or more cancer diagnoses
19	Type of surgery
20	Chemotherapy
21	Radiation therapy
22	Hormone therapy
23	Immunotherapy
24	Treatment with Trastuzumab
**Values**	
25	Intention to RTW
26	Meaning of work
27	Work attachment
28	Sense of professional usefulness
**Physical/Psychological factors**	
29	Global health status
30	Physical fatigue
31	Cognitive fatigue
32	Emotional fatigue
33	Disability due to BC
34	Pain
35	Physical sequelae
36	Restricted arm movement
37	Body image
38	Emotional distress
39	Depression
40	Anxiety
41	Post-traumatic growth
42	RTW self-efficacy
43	Benefit finding
44	Problem-focused coping
45	Emotion-focused coping
**Work environment**	
46	Physical stressors at work
47	Psychological stressors at work
48	Organizational stressors at work
49	Work-related stress
50	Perceived social support from colleagues
51	Perceived social support from managers
52	Recognition by colleagues of the quality of work performed
53	Recognition of the BCS by line management in her professional activity
**Medical environment**	
54	Quality of the met supportive care during BC treatments
55	Perceived social support from medical staff
56	Early liaison with occupational practitioner or occupational health department
57	Social worker support
**Personal environment**	
58	Perceived social support from family
59	Perceived social support from friends
**Therapeutic margin of manoeuvre**	
60	Working time accommodation
61	Workstation accommodation
62	Professional duties accommodation

### Development of an integrative and transactional conceptual model

**Fig. 2:**
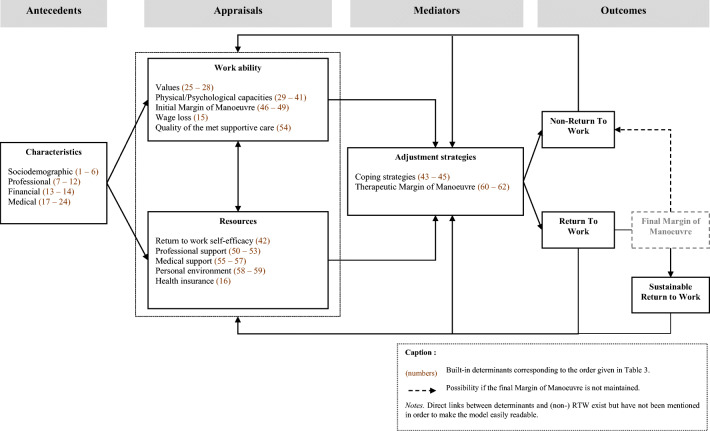
An integrative transactional conceptual model of the RTW of BCS (REWORK-BC model)

Six of the 11 experts recruited for the TRIAGE exercise made themselves available to comment on the conceptual model (Table 1 — experts 1, 2, 7, 8, 9, and 11). The positioning of the concepts within the different categories of the conceptual model was validated by all. The conceptual model presented included, in detail, the 62 determinants identified by the TRIAGE method. All experts consulted proposed that the determinants be grouped into subcategories to improve readability. The titles of each category and each subcategory of the conceptual model were only discussed with experts 1, 2, 8, and 9 (Table 1). The final conceptual model comprises the BCS’ characteristics and the broad categories of the transactional process such as primary appraisal (work ability), secondary appraisal (resources), adjustment strategies, outcomes (RTW/non-RTW), and feedback (Fig. 2). Each category of the model includes the several determinants supported by the expert consensus.

#### Antecedents: BCS’ characteristics

BCS’ characteristics included (i) sociodemographic factors (Table 3 — items 1 to 6); (ii) professional factors (Table 3 — items 7 to 12); (iii) financial factors (Table 3 — items 13 and 14), and (iv) medical factors (Table 3 — items 17 to 24). BCS’ characteristics have been associated in the literature with the patient’s appraisal of her work ability and resources [38].

#### Primary appraisal: work ability

Work ability refers principally to physical and psychological capacities in relation to work demand [39]. The primary appraisal consists of a global evaluation of physical/psychological capacities (Table 3 — items 29 to 41), financial situation (Table 3 — item 15) according to the worker’s expectations, and their intention to RTW (i.e., work values, Table 3 — items 25 to 28), initial MM (Table 3 — items 46 to 49), and the quality of the met supportive care during treatment (Table 3 — item 54).

The initial MM assessment according to individual values and physical/psychological capacities allows RTW to be identified as a challenge, a loss, or a threat as recommended in the transactional perspective [24]. There are four scenarios if the patient intends to RTW according to the relationship between physical/psychological capacities and the initial MM:

- Physical/psychological capacities and the initial MM are both high: RTW is conceivable and therefore becomes a challenge.
- Physical/psychological capacities are low with a high initial MM, and the patient feels a strong personal and/or financial need to RTW: RTW is conceivable and therefore becomes a challenge.
- Physical/psychological capacities are high, but the initial MM is low: RTW may generate a loss of physical/psychological capacities favoring the development of physical and psychological disorders (e.g., fatigue, burnout, emotional distress, musculoskeletal disorders).
- Physical/psychological capacities and the initial MM are low: RTW represents a threat rendering it impossible in practice.

This primary appraisal represents all the information that can be collected when the BCS is considering RTW. It is an individual assessment of the situation which must also take into account the resources available to facilitate the RTW.

#### Secondary appraisal: resources

The resources appraisal describes the perceived dimensions of work ability. Resources may act as positive or negative moderators of the relationship between work ability and RTW. Five types of resource were identified: RTW self-efficacy (Table 3 — item 42), professional support (Table 3 — items 50 to 53), medical support (Table 3 — items 55 to 57), personal environment (Table 3 — items 58 and 59), and health insurance (Table 3 — item 16).

By means of a dual appraisal, of work ability and resources, it will then be possible to implement relevant and effective individual or professional strategies to facilitate the RTW.

#### Strategies required to facilitate RTW: adjustment strategies

Adjustment strategies refer to the implementation of all the strategies that are necessary for RTW according to the specific needs of the BCS. For BCS with physical/psychological impairments, effective coping strategies (Table 3 — items 43 to 45) need to be developed. According to the transactional perspective, the effectiveness of coping strategies depends on the BCS’ level of RTW self-efficacy [24]. Self-efficacy is one of the two components of perceived behavioral control [40]. The other dimension, perceived controllability [40], is less relevant since RTW does not depend solely on the patient but also on the work situation and a set of stakeholders [10]. The higher the RTW self-efficacy level, the more effective the problem-centered coping strategies are. Conversely, the lower the RTW self-efficacy level, the more effective the emotion-centered coping strategies are. For BCS whose work situation does not favor a RTW, it is necessary to implement accommodations with respect to working time, workstation, and/or professional duties conceptualized as “therapeutic MM.” For yet other BCS, it is necessary to increase coping skills while putting in place a therapeutic MM (Table 3 — items 60 to 62).

#### Outcomes and feedback

Whether the RTW process succeeds or fails, dynamic re-evaluations take place over time to either sustain the RTW or implement the required modifications in the event of non-RTW. Following a successful RTW, the objective is to keep the adjustment strategies in place to promote a sustainable RTW, conceptualized as the final MM.

## Discussion

The aim of this study was to propose a conceptual model to explain the BCS’ RTW process. Identified determinants, according to a consensus of experts, corroborate the results observed in previous studies [4, 33, 35–37]. Of the conceptual models available in the literature [8, 9, 13, 17–19, 41–43] that explain the relationship between cancer survivorship and work, only two focus on BCS’ occupational outcomes [13, 17], whereas RTW after cancer is highly dependent on tumor location [44, 45]. The REWORK-BC model (Fig. 2) was designed specifically to explain the RTW process of salaried workers diagnosed with BC according to transactional theory [24]. However, it could be adapted to other cancer pathologies by removing the BC-specific characteristics (e.g., arm movement restrictions). Cultural adaptation of the current model might be necessary as policies, procedures, and economic factors may influence social context, work environment, or climate, which could have a major impact on the RTW of patients diagnosed with cancer [42].

Previous biopsychosocial models that explain the BCS RTW process focus mainly on the individual determinants of RTW without integrating the dynamic aspect of the process or the environmental components related to the occupational and care dimensions [13, 41, 42]. In addition, the transactional-based model proposed by Brusletto et al. [9] is relevant to the understanding of the five phases of the RTW process (1 entering the world of cancer; 2 fighting for life; 3 fighting for work and renewed normality; 4 creating a new reality; 5 sustainable work in one’s new reality). However, this model does not explain in an integrative way the links (direct or indirect) between the determinants of the RTW process as advocated by Knauf and Schultz [14]. The transactional perspective, underlying the REWORK-BC model, allows consideration of the dynamic aspect of the process by integrating both the interactions between the primary and secondary appraisals and transactions between these appraisals and mediators to cope with the situation [24]. In addition, the REWORK-BC model integrates several systems making it ecologically valid and generalizable [14]: (i) the individual system specific to the BCS’ characteristics and perceptions, (ii) the hospital system including the role of health professionals, (iii) the occupational system including the role of managers and colleagues, and (iv) finally the financial system. The parsimonious and multidisciplinary aspects of the REWORK-BC model — combining concepts of health psychology, rehabilitation, and ergonomics — make it workable for clinical practice. Direct and indirect relationships between the components of the model were clearly explained, and all the variables included are easily measurable using validated tools available in the scientific literature.

Swanberg et al. [43] developed a five-system cancer work management process using an ecological perspective describing the influence of interrelated systems on human development [46]. While relevant, this model does not provide the interdisciplinary and multifactorial perspective expected to explain the RTW process after cancer. Each step in the REWORK-BC model helps to identify the determinants on which patient-centered interventions can act to promote RTW, making it effective. (i) The assessment of antecedents allows the early identification of women at risk of non-RTW [42], permitting the setting up of liaison with occupational health specialists at an early stage to plan RTW supportive care ahead of time. (ii) The primary appraisal (work ability) determines whether the RTW process will be a challenge, a loss, or a threat for the patient. It will allow healthcare professionals to establish an initial intervention plan that matches the patient’s intention to RTW by considering physical, psychological, and/or financial needs according to the patient’s work situation [39]. (iii) The secondary appraisal (resources) will allow an assessment of the individual resources, the professional resources, the medical resources, and the financial resources available to the patient. Put into perspective with the primary appraisal, this will allow identification of the individual, ergonomic, managerial, and environmental triggers. (iv) This model integrates all individual effort and accommodations (therapeutic MM) deployed to cope with the stressful situation [24, 25, 28]. (i) Firstly, BCS (non-)RTW if they perceive (or do not perceive) a benefit in doing so (i.e., a benefit-finding strategy) [47]. Secondly, patients can implement problem-centered or emotion-centered strategies that mediate the relationship between appraisals and (non-)RTW; (ii) a therapeutic MM is a key component that refers to the set of ergonomic provisions implemented to adapt the work situation to the BCS’ work ability [48, 49]: professional duties and tasks, working time, ergonomic characteristics of the workstation, etc. Through the mediating effect of these work accommodations, RTW may be possible despite an unfavorable health condition.

### Implications for BCS

The REWORK-BC model is beneficial to BCS in that it views them as active participants in their RTW process. The identification of the 62 determinants associated with the RTW of BCS will help to design tools that can be used easily by all stakeholders involved in the RTW process. Moreover, the REWORK-BC model will help with the development of multidisciplinary interventions including medical, psychological, social, financial, professional, and ergonomic supportive care. This will allow the systematization of the assessment of the RTW determinants by clinicians to personalize interventions with the aim of increasing their effectiveness over time. These interventions must be implemented according to the specific needs of each patient at each stage of the model ensuring that the BCS is seen as a person with freedom of choices. Indeed, each person is unique and can always make personal choices that can give good results against all odds.

### Limitations and strengths

A limitation inherent in the TRIAGE method pertains to the group dynamic. Some experts, considered to be recognized scientific experts, may influence the opinion of other experts such as BCS included in the group. To counterbalance this effect, the moderator ensured that everyone had a fair opportunity to express himself/herself. The final voting, after the discussion, was also conducted on an individual and anonymous basis to ensure that everyone’s opinion was respected. In addition, a review of reviews was performed which allowed the listing of the possible determinants of the RTW of BCS. The objective was to prepare for consensus and not to report on a systematic review of the literature. Combining the results identified from the literature and the expert opinions ensured however the completeness and relevance of the selected items.

Only two BCS were included in the consensus, one of whom is a Head of a patient association. A third BCS, who had initially agreed to participate in the consensus, was ultimately not available for personal reasons. A close caregiver (e.g., spouse or cohabitant) representative also did not take part in the consensus exercise. Additional information concerning the value of family relationships might be considered in the RTW process of BCS. The inclusion of different stakeholders (e.g., healthcare professionals, BCS, lawyers, human resources specialists, researchers) with varied expertise in the fields of cancer, and RTW, is however a major strength of the study. This resulted in a multidimensional approach based on the scientific literature, clinical and personal experiences, and practices. The participative approach of the TRIAGE methodology, namely the comparison of opinions in the collective consultation phase, led to a shared perspective on the issue of the RTW of BCS. Another strength lies in the proposal of a new dynamic model of the RTW of BCS that complies with the recommendations of Knauf and Schultz [14].

## Conclusions

There is a need to better understand the articulation between the factors involved in the BCS RTW process and to develop integrative and operative BC-specific approaches explaining the RTW process. Based on knowledge, experience, and clinical practices, the REWORK-BC model includes the medical, psychological, social, financial, professional, and ergonomic aspects of the RTW of BCS. To the best of our knowledge, this is the first systematic interdisciplinary dynamic model of the RTW of BCS. It is capable of further development, for example, for cancer patients with pathology in other types of location. This integrative, operational, and transactional conceptual model of the RTW of BCS will help to design more efficient patient-centered intervention studies.

## Abbreviations

BC: Breast cancer
BCS: Breast cancer survivors
MM: Margin of Manoeuvre
RTW: Return to work
TRIAGE: Technique for Research of Information by Animation of a Group of Experts
